# Reconfiguring Social Value in Health Research Through the Lens of Liminality

**DOI:** 10.1111/bioe.12324

**Published:** 2017-01-06

**Authors:** Agomoni Ganguli‐Mitra, Edward S. Dove, Graeme T. Laurie, Samuel Taylor‐Alexander

**Keywords:** social value, health research, liminality, bioethics

## Abstract

Despite the growing importance of ‘social value’ as a central feature of research ethics, the term remains both conceptually vague and to a certain extent operationally rigid. And yet, perhaps because the rhetorical appeal of social value appears immediate and self‐evident, the concept has not been put to rigorous investigation in terms of its definition, strength, function, and scope. In this article, we discuss how the anthropological concept of liminality can illuminate social value and differentiate and reconfigure its variegated approaches. Employing liminality as a heuristic encourages a reassessment of how we understand the mobilization of ‘social value’ in bioethics. We argue that social value as seen through the lens of liminality can provide greater clarity of its function and scope for health research. Building on calls to understand social value as a dynamic, rather than a static, concept, we emphasize the need to appraise social value iteratively throughout the entire research as something that transforms over multiple times and across multiple spaces occupied by a range of actors.

## 1 Introduction

The concept of social value has a long history in research ethics, appearing in one iteration as early as the Nuremberg Code – ‘The experiment should be such as to yield fruitful results for the good of society’^1^ – and has grown in stature in contemporary literature^2^ and bioethics policies. In the United Kingdom, for instance, research ethics committees are expected to consider how a given research protocol ‘enables ethical and worthwhile research of benefit to participants or to science and society’,^3^ and to consider how the ‘benefits of research evidence for improved health and social care, should be distributed fairly among all social groups and classes.’^4^ Social value also appears prominently in the recently proposed revisions to the International Ethical Guidelines for Biomedical Research Involving Human Subjects from the Council of International Organizations of Medical Sciences (CIOMS). CIOMS Guideline 1 calls for making social value an explicit part of the ethics evaluation and approval by researchers, research ethics committees, regulators, and sponsors. As it states in part: ‘The ethical justification of health‐related research involving humans is its social value: the prospect of generating the knowledge and/or the means necessary to protect and promote people's health.’^5^

And yet, perhaps because the rhetorical appeal of social value appears immediate and self‐evident, hitherto the concept has not been put to rigorous investigation in terms of its definition, strength, function, and scope. Indeed, in the bioethics literature, social value has been employed as a placeholder for a plethora of ideas, including: the importance of research; the relevance of research; the validity of research; clinical value and health value;^6^ an ethical benchmark for practices as narrow as specific interventions;^7^ a measure against frivolous use of resources and of exploitation;^8^ and a proxy for concepts as broad as generalizable scientific knowledge.^9^

No doubt social value has a central role to play in the ethical acceptability of health research, but might we come to some descriptive and normative assessment of its essential characteristics? In this article, we discuss how the anthropological concept of liminality can illuminate social value and differentiate and reconfigure its variegated approaches. Employing liminality as an analytic and normative frame encourages a reassessment of how we understand the mobilization of ‘social value’ in bioethics. The utility of this frame lies in revealing the *stages* that unfold in health research and the *multiple thresholds* that must be crossed to achieve the delivery of the public good that is scientifically sound and ethically robust health research. Liminality can reveal how the appeal and functions of social value change throughout these processes as they unfold, thereby necessitating on‐going assessment.

We argue that social value as seen through the lens of liminality can provide greater clarity of its function and scope for health research. Building on calls to understand social value as a dynamic, rather than static, concept, we emphasize the need to appraise social value iteratively throughout the *entire* research lifecycle, from the research design stage through publication and dissemination of research results, to data storage and sharing for future research. Social value is, as a result, something that transforms many times and across many spaces occupied by a range of actors. In other words, we see social value as undergoing relatively constant change across all *limens* (thresholds), as research unfolds and as the ‘social’ is assembled and re‐assembled along research pathways.

The remainder of this article is divided into five sections. First, we provide a taxonomy of how social value is employed in the bioethics literature. We then describe the anthropological concept of liminality and show how using the lens of liminality can illuminate many of the extant conceptual issues with social value. Thereafter, we offer examples to illustrate the potential advantages of re‐visiting the processes of research and social value through this lens, while also reflecting on possible disadvantages. Normatively, this culminates in a schema of five elements (or queries), that are inspired by this heuristic and that can be applied to health research endeavours to evaluate their evolving social value iteratively, robustly, and coherently. Finally, we propose the example of meaningful patient and stakeholder engagement as a paradigm instance of how social value can be assessed in such terms.

## 2 Social Value in Research Ethics

In order to develop the analytic and normative frame of liminality, it is important to consider first how the concept of social value is currently employed in bioethics. From an overview of the current literature and discourse, we suggest that the concept performs three key — sometimes implicit — normative roles. First, social value is employed as a *teleological* device: social value is the ‘end’ of research, while research itself has instrumental value.^10^ Here, social value is appealed to as the primary justification for, or fundamental meaning behind, engaging with the research endeavour in the first place.^11^ Second, social value is employed as a *threshold* device: it is used as an evaluative criterion to usher a research proposal over the early‐stage ‘threshold’ of ethics approval. Within this reading of social value, two normative appeals are frequently found: (i) to justify resource allocation *to* research,^12^ and (ii) to justify the risk and burdens associated *with* research.^13^ Third, social value is employed as a *protective device*: research which does not have social value is deemed exploitative – directly of participants^14^ (because risks are otherwise not justified) and perhaps also of society (because indirect risks and resource use are not otherwise justified). This appeal aims at preventing research and its actors from taking unfair advantage of individuals and of society. Importantly, these three roles are not mutually exclusive. Indeed, an appeal to social value can be an appeal to perform all roles, as it does in the proposed revisions to CIOMS.^15^

While all three roles of social value are crucial to delivering the public good of health research, the specific work required of social value in the discharge of such roles at various junctures in the research process has not received adequate attention. As we explain below, a liminal framing helps us to trace the changing nature of these roles across and through the ethics approval and research landscapes. This not only allows us to understand better the respective roles for social value, but it also assists in evaluating social value as part of the research process itself.

## 3 Social Value in Light of Liminality

### Why Liminality

The anthropological concept of liminality draws attention to process and transition from one stage to another. This focus on process and transition emerged through early ethnographic research of ritual practices that sought to understand social transformation. In the early 20th century, the French anthropologist Arnold van Gennep identified liminal rites as an important part of the *reproduction of social order*.^16^ Positing a tripartite model, van Gennep's schema outlined: (1) the symbolic and spatial separation of an individual from their existing social position (pre‐liminal); (2) the transformation of their social status as they pass through an adjacent, often marginal space that is characterized by a dissolution of established social order and hierarchy (liminal or ‘threshold’); and (3) their spatial and symbolic reincorporation into society (post‐liminal).^17^ Because the suspension of social order is spatially and temporally limited, such ritual practice allows for social transformation to occur in a manner that preserves broader organizational structures.

Crucially, van Gennep's well‐evidenced claim was that liminality permeates all societies, and to witness it can help us understand processes of transition and transformation in many areas of life.^18^ Classic examples are the transitions from childhood to adulthood, from wellbeing to ill health, and from ill health to death. If liminality is about identifying significant thresholds – and *asking what happens beyond and between those moments, both for the individuals involved in a ‘rite’ and the broader social order* – then this encourages an interpretation of the ‘rite’ of ethics review of a research application as but one (albeit a critically important one) of a number of thresholds that are confronted and often crossed in the research lifecycle.

To continue the metaphor of the ‘rite of passage’ of ethics approval, once this first threshold is crossed, significant transformations then occur. Most particularly, a research protocol transitions *from a mere proposition of involvement with participants to an actual plan of action with participants*. This implicates a range of actors, and importantly, it further transforms individuals (be they healthy ‘volunteers’ or patients) into active research participants.

### Beyond preliminary thresholds

Demonstrating the social value of the research protocol (and attendant documents) forms part of the criteria that must be met to allow this initial transition to take place. In other words, social value here is used as a preliminary threshold device. Once a research project is given ethics approval, however, it moves from a pre‐liminal to a liminal phase. But the evaluative process does not end here. In fact, a liminal framing suggests that we must follow this process through because the research endeavour then enters a new phase with different implications and actors (such as recruitment of patients, involvement of research nurses and clinicians, or the need for regulatory approvals). Moreover, liminality is a temporary condition — we pass *through* liminality and emerge from the process. This requires us further to consider the *telos* of the research process. The liminal phase itself is mediated by ethical, medical, and scientific norms, and crucially, it often involves adjustment and serendipity as researchers encounter both problems with research design and unexpected findings.^19^ This suggests that social value in research always plays a role *beyond* its use as a preliminary threshold device. As Emanuel and colleagues state:

Clinical research is not an end in itself. It has instrumental value because it generates knowledge that leads to improvement in health or health care. It is such improvements in health that ultimately constitute the social value of research.^20^

The apparent tension arising from using both a broad, long‐term appeal to social value alongside a narrow and immediate social value requirement arises from the fact that *social value performs both a teleological and threshold function simultaneously*. This tension partly exists as a result of the inherent uncertainties of research. Emanuel and colleagues call for social value to be a one of eight criteria for determining whether research is conducted ethically, but they rightfully acknowledge the uncertain nature of both social value and research, even of otherwise rather specific research protocols:

Priorities may change while a study is being conducted, and the cooperation of diverse groups is often needed to make changes based on research results. This makes the process of going from research to health improvements uncertain and arduous. Assessment of the value of research is made prospectively before any data are collected. Consequently, determinations of social value are uncertain and probabilistic, entailing judgments about the usefulness of a sequence of research and chances of implementing the results. Even in wealthy countries with well‐established research studies and health system infrastructures, research results are imperfectly incorporated into clinical practice.^21^

We would add that the uncertain and probabilistic nature of social value is an illustration of the inherently liminal nature of research. We suggest further that viewing research as a liminal process for researchers and participants alike means that the nature and content of what is considered – or should be considered – social value evolves, and rightly so. The lens of liminality, in further emphasizing transition and thresholds, focuses our attention to those points in the life cycle of research when, for example, a study or results, and their associated value, were once one thing and are becoming something else. This also requires to us to be further attentive to the relevant actors, processes and interests associated with those transitions since all of these might change as a result of crossing a significant threshold. For example, an archive of brain scans may have been established with a specific idea of how these are socially valuable, but their value could change as new discoveries are made about biological markers of dementia, or by linking this archive with participants’ medical records over time. The archive itself is in a liminal state, potentially taking on new and different value over time. Liminality therefore demands recognition that ‘the social’ (comprised of various actors operating in various networks) undergoes constant reassembly; the ‘social’, as much as ‘value’, struggles to be evaluated as a static entity. Any attempt to fix social value at an early point in the research process is flawed except as an unproved promissory pre‐liminal claim. Liminality suggests that matters can and will change, including the nature and value of social value itself.

### The evolving nature of ‘value’

The multiple, processual changes in research create new avenues for value, new entities that are valuable, new actors to generate or steward value, new populations to whom value may accrue, and new pathways for generating further social value. Consider, for example, how the value of experimental therapeutics and potential vaccines for Ebola virus disease suddenly increased by several orders of magnitude as the spread of the virus became a public health emergency in the face of frail health systems unable to contain it. The lessons learned from Ebola have directly influenced the ways in which the WHO is considering R&D for the more recent Zika virus outbreak, establishing frameworks and coordinating activities with the industry and groups studying medicinal responses to Zika.^22^

Applying this insight to social value, we see that ‘the social’ is constantly in formation. It can be thought of as an unstable, if not unstructured, assemblage of different components that modifies and is modified as research unfolds. The *value* of a given research project is also ‘in‐the‐making’ during the liminal phase. It is modified along with the social as, for example, the downstream applicability of the new knowledge becomes clearer. Put simply, the heuristic of liminality alerts us to this *transformational* dimension of research and thus requires us to rethink how the coupling of the social and value into ‘social value’ is assessed during ‘rites’ in the research lifecycle, such as ethics review and protocol milestones, and how it is assembled and reassembled in the processes of research itself.

This is illustrated in some recent approaches to research ethics. For example, Rid and Wendler have developed a framework aimed at improving the risk‐benefit evaluation of research that is closely associated with social value as a normative concept.^23^ Touching on all three roles of social value as described above (teleological, threshold, and protective), Rid and Wendler suggest that an adequate risk‐benefit evaluation, coupled with social value, is essential in justifying research, protecting professional integrity of researchers and maintaining public confidence.^24^

Interestingly, however, while social value is explicitly used as a threshold device in Rid and Wendler's approach, they also suggest ways in which consideration for future research, such as specimen storage, might affect early‐stage considerations about risk.^25^ Moreover, while considerations about social value are meant to arise at the earliest stages of research, Rid and Wendler introduce them along with open questions, for example, with regard to the definition and scope of the concept, as well as the actors involved in such decisions. We would concur. The clear necessity of introducing a concept as dynamic as social value to what tends to be considered a rather structured or evident part of ethics approval, points to the need to engage not only with the analytic implications of the concept, but also with its normative and regulatory implications over time. We suggest that the explicit recognition of liminality in research ethics and regulation help us to do so.

In summary, health research is confronted with, and must overcome, multiple thresholds: from research design, to ethics approval, to participant recruitment, to data generation, to analysis and research findings, to publication, to knowledge translation, and so on. As we have seen, appeals to social value often aim past these thresholds and simultaneously employ social value as the *telos* of these processes, or the ‘end’ of research, in the form of (benefit‐enhancing) generalizable knowledge. Liminality encourages us to identify and pay attention to these symbolically and practically significant thresholds. It requires us to focus on the need to evaluate and re‐evaluate social value at each threshold, which are likely to contain a different mix of actors and considerations, while always keeping in mind its teleological role in the assessment of research value. Furthermore, the preliminary and promissory appeal to social value ought to be recognized as such; delivery of a materially different set of social values from research ought not, therefore, to be automatically adversely judged so long as these generate actual value to the ‘social’ as constituted at the relevant time, and engaging the relevant actors.

A pertinent example to illustrate this comes from the responses to the West African Ebola virus epidemic that occurred from 2013 until 2015, which we highlighted above. Very quickly into the epidemic, ethical questions arose around emergency use of unregistered experimental interventions, and justification for vaccine trial design that had implicit, but strong, association with the evolving nature of social value.^26^ Interventions that were formerly solely in the experimental domain (unproven for safety and efficacy in human beings) suddenly crossed a value‐threshold into the domain of potential ‘emergency use’ (provided that data from their use were systematically collected and shared)^27^. The establishment of MEURI^28^ (monitored emergency use of unregistered and experimental interventions), one example among many urgent considerations at the time, disturbed the traditionally established importance of, and distinctions between stages of research, between care versus research‐obligations, the value of randomized controlled trials, and the very telos of research.^29^ It was suggested for example, that individually randomd placebo‐controlled trials might not be acceptable to the communities in question^30^. In other words a community, under such disaster conditions, may put far more emphasis on the social value of unproven (and therefore risky) but potentially therapeutic interventions, than on existing standard and supportive care. Public health emergencies such as this are an excellent, if unfortunate, example of why social value needs to be evaluated over time (and even re‐evaluated) by RECs, data monitoring committees, and other actors, as its scope and strength – what is valuable? How valuable is it? – might be recast in various lights during a health emergency. Indeed, it has been argued that much research carried out during emergencies such as Ebola, would necessarily make considerations such as risk or social value ‘shifting targets’ of ethical assessment.^31^

## 4 Normative and Regulatory Implications

Research never happens in isolation from existing socio‐political values and institutions, healthcare systems, policies, and markets.^32^ The concept of value, being inherently normative, is to a large extent context and actor‐dependent. Not only does the question arise: ‘valuable to whom?’, but so also do the questions of: ‘valuable as determined by whom?’, and, ‘by what measures?’ Research is engaged with ‘multiple orders of value’^33^ and sits amid a variety of conflicting values and interests, not to mention power structures. The proposed revised CIOMS Guidelines, for example, suggest that sponsors, researchers, regulators, and research ethics committees must all ‘ensure’ that the principle of social value is met,^34^ but the Guidelines do not explain in what ways these actors should discharge such a duty, nor whether the duty continues downstream (and might branch out to other actors), nor whether it is based on the ‘tangible value’ from the research results or the ‘promised’ value from the preliminary ethical approval stage. The challenge is that at the moment of evaluation, the question of social value is far beyond the control of many of the very actors whose duty it is to ‘ensure’ social value. As Habets and colleagues observe:

Whether a particular research *direction* has sufficient ‘value’ is at the moment decided by the research funding agencies, steered by political decisions. Although the public in a democracy has thus an indirect voice in the research agenda, it can be questioned whether there is enough transparency in the decisions made. Funding agencies determine priority by constructing research programs, within which calls for grant proposals are made.^35^

Under such conditions, determining or indeed ‘ensuring’ the prospects of social value are tasks that are both challenging and politically contestable. It is not inconceivable that social value as an ethical requirement or principle runs the risk of excluding projects which, based on a preliminary assessment, ask broad (academic) questions that do not necessarily have a direct or immediate impact on health, or that are critical of underlying dominant socio‐political structures. Yet, under the broader, more flexible interpretation, and conceding that research is a public good, it could be said that all research has *some* social value, regardless of who determines (or ensures) its prospects. Just as broadly, the concept of social value has been said to protect not just participants, but also ‘public confidence in the research endeavour’,^36^ and to provide ‘credible social assurance’.^37^ Such diverse teleological uses of social value within the inherently uncertain processes of health research, aimed at a multiplicity of actors, could generate considerable ambiguity for actors about the precise standards to which they will be held to account.

In light of the above analysis, we do not suggest that a ‘gold standard’ approach is possible or viable across a wide range of health research, but the examples offered herein do nonetheless evoke elements of the construction of social value that reflect many of the realities of health research as an essentially liminal process. We suggest that the following five elements and queries can be extracted from our analysis when seeing social value in these liminal terms. Each is informed by the focus brought about by the liminality lens, namely, its emphasis on process, thresholds, and transformations over space and time. Thus, we have considerations that relate to:

- **Temporality**: what timeframes, and which thresholds, are likely to be involved across the entire research lifecycle? How does social value inform stages in the lifecycle and, and at the same time, how is it transformed by these thresholds?
- **Spatiality**: which actors are implicated in which spaces within the relevant timeframe, and who is likely to contribute to the construction of social value at each timeframe? How do actors and actions operate to take research across the thresholds and how are these processes informed by social value?
- **Validity**: how well do appeals to social value (particularly by researchers) accurately reflect the presence or absence of value, produced over time? Does the validity of social value as constructed in the *pre‐liminal* (research approval stage) change as research gets underway and evolves, and what does this mean for oversight?
- **Reliability**: who can expect to benefit from social value(s), as actually produced, and in what ways?
- **Accountability**: who must give account for identifying social value(s) from these processes, and how? Who is liable if ultimate social value in the guise of tangible social benefit is not realized?

This schema can be applied by different actors (such as ethics committees, researchers, and policymakers) to various health research endeavours, including more ‘discrete’ projects such as clinical trials that still nevertheless undergo multiple stages across the research lifecycle (from initial approval to recruitment and consent, to data collection and dissemination and so on). To consider a relatively ‘easy’ example: UK Biobank (UKB) is a long‐term biobank study with 500,000 participants; it has an Ethics and Governance Council (EGC) that acts as a critical friend and supports the design of effective policies on its long‐term operations and success. The EGC operates in a *reflexive* way such that UKB and its policies are constantly re‐evaluated and approaches to research and participation are adapted to assure they deliver on their stated objectives, as outlined in the UKB Ethics and Governance Framework (EGF). The EGF is determinedly a living instrument that embodies the obligations of the researchers to participants and wider society. The EGC advises on necessary changes to this document over time, and the entire process allows for the accommodation of changing notions of social value. This is facilitated by broad consent from participants ‘to participate in UK Biobank’ where it is made explicit that UKB exists to support ‘health‐related research’.

In contrast to regulatory approaches in more traditional research practices, relying either on law or regulations to enforce norms of behaviour, many research biobanks take a ‘complex systems approach’ to ethical evaluation of genomic and other health research. As one of us has observed, this ‘is typified by research governance policies which promote openness and sharing (communalism), population‐based participation in research (citizenry) and joined‐up initiatives to realize the research promise (convergence).’^38^ These are all instance of social value, albeit unspecified.

Admittedly, contemporary research biobanks are well suited to a liminal approach to social value as they are constituted to support a wide range of future research projects. Many have robust governance processes designed to adapt to changes in social value. Yet a liminal approach can be applied as robustly to other areas of health research that demonstrate the fluidity and multiple stages in the lifecycle where social value can change and undergo reassessment.

Consider the example of Guthrie card collections set up in many Western states in the 1960s. Initially designed to test newborns for treatable conditions such as congenital hypothyroidism, the retention of the blood samples on cards in state‐held collections numbering millions now means they represent a potentially very valuable genetic research resource. Many such collections never envisioned such uses; indeed, most cards obtained in the early period will not have any specific (let alone informed) consent. The social value in research terms was simply not envisioned at set‐up. But what started as a matter of an individual's early clinical record has transformed over time into a biomedical collection — of little or no clinical value — probably held and stewarded today by very different professionals under very different conditions. As a recent report has pointed out:

The transformative potential of biomedical collections for individual, local and global health is exponential. Scientific and technological advances mean that the possible future uses of the Guthrie collection are constantly changing and these no longer depend only on developments in the health sector – cloud computing and mobile applications mean that these valuable resources can be enriched and shared in ways never before contemplated. This also raises the possibility of a far more engaged role for the citizen interested in contributing to and influencing the future direction of research.^39^

Some Guthrie card collections have been destroyed for want of a demonstrable social value.^40^ Others require urgent attention to the legal, ethical, and governance issues that can bring about the potential value that they hold. This process itself could be assisted considerably by recognizing these collections as existing in a liminal phase, and by addressing our five elements to elicit the precise role that social value might play in their continued existence.

These examples, along with CIOMS’ renewed emphasis on social value, evidence the need to ‘follow’ a research project along its discursive paths and trace the evolution of social value across all thresholds, paying attention to elements such as temporality, spatiality, and accountability. A narrow reading of social value would fit well within a regulatory paradigm that thrives on certainty and protecting individual rights and interests – as much of law does – but it fits poorly within a paradigm that reflects the nature of health research, that is, one that deals in uncertainty, complexity, and dynamism, and seeks to promote trust and the public interest as a means of satisfying the public good of research. The normative schema inspired by liminality encourages both on‐going reflection of the nature and scope of social value and also, as we explain below, understanding that both the ‘social’ and ‘value’ are dynamic concepts best elucidated in a reflexive and inclusive manner.

Such a dynamic approach to thinking about and using social value is best expressed when we shift our epistemological frame of social value as a regulatory paradigm – as a *rule* for what ethics committees must look for and ensure at the point in time when they receive submitted materials from a researcher – to a liminal paradigm, which can help reconfigure the approaches to social value to take account of processual factors. A liminal approach attuned to the elements and queries identified above means that we cannot simply ‘assume’ the social, tacking on the adjective to the noun ‘value’ as though the social simply ‘is’. As Bruno Latour reminds us:

When social scientists add the adjective ‘social’ to some phenomenon, they designate a stabilized state of affairs, a bundle of ties that, later, may be mobilized to account for some other phenomenon. There is nothing wrong with this use of the word as long as it designates what is *already* assembled together, without making any superfluous assumption about the *nature* of what is assembled.^41^

Here, then, social value is better envisioned as a loose form of a social covenant (or bond) whereby those involved in a research project pledge in good faith to make a reasonable attempt to deliver an array of potential benefits for an array of potential beneficiaries, with a willingness to re‐evaluate what the value is or may be, and its delivery, as the research unfolds. This covenant necessitates constant reflection and adaptation, along with changing understandings of the value that may accrue, and to whom it may be of value. This, in turn, requires an inclusive understanding of who may contribute to, or indeed help guide, such dynamic and on‐going reflection.

At the same time, this processual account raises important questions about accountability for the plethora of potential forms of social value. We remind the reader that liminality is about passing through, *and emerging from*, a transformational phase of human experience. As such, it is crucial both to be open to what might count as social value arising from this process, and also to consider who must account for potential social values that in turn emerge, and how this is to be done. This is not the same task as realizing social value from research. Consider, again, the Guthrie card example: even if such collections could yield new generalizable knowledge, their continued storage and use as a de facto DNA database with heterogeneous or non‐exist consent raises crucial social questions about legitimacy and accountability. This illustrates very well the imperative to clarify responsibility to follow social value through these processes, in whichever forms this might take.

Another implication of social value seen through the lens of liminality is its impact on the work of ethics committees. As we have stressed, such a perspective would encourage ethics committees (and the regulatory frameworks which govern them) to reassess social value further downstream and not just at the initial stage when an application is first submitted for ethical review. For example, committees would be attuned to social value implications when considering interim or final reports tabled by research teams, and when assessing researchers’ dissemination plans. Depending on the answers we give to the normative schema developed, or that we expect to give to the questions of: temporality, spatiality, validity, reliability, accountability, we may want to develop different kinds of research oversight. For example, if some of the central concerns about validity and accountability around open ended uses of existing data or the repurposing of an existing drug for a specific new use – whose risk repercussion is considered minimal – are contemplated, then a reconsideration of social value could be informed by interim ethics review. This could include specific plans to revisit the assessment of social value further down the line, and could be an extension of the initial up‐front ethics review within the research lifecycle. In contrast, other types of interventions, such as research conducted during public health emergencies – under conditions of considerable uncertainty – might benefit from a model akin to clinical ethics, with bespoke, embedded and on‐going oversight. These are very different processes for attempting to bring about novel social value in the face of a spectrum of known and unknown risks. Accordingly, the changing nature of social value throughout these diverse research endeavours could have any number of implications: it could encourage further investment, changes to trial design or patient/participant recruitment, or indeed warrant putting a stop to certain types of research if an ethics committee (or other appropriate body) deemed the research (presently and foreseen) no longer to contain any social value. Our point is that full and responsible engagement with the shifting dynamics of social value across heterogeneous research processes maximizes the chances of delivering benefit while reducing risk and allowing research to flourish.

An integral part of this responsible process would require oversight bodies also to assess the social value implications of researchers’ stakeholder engagement plans that set out which ‘actors’ will be engaged to co‐design the research or serve in another capacity. This would include assessment of the significance of preliminary results and any substantial amendments to the research protocol. Not only would this likely strengthen protections afforded to research participants, it would also encourage greater stakeholder engagement, which we discuss further below.

We admit that there are some potential downsides to conceptualizing social value through the lens of liminality. For example, iterative re‐evaluation of the value of projects by multiple actors could be practically infeasible for a variety of actors, be they ethics committees, data monitoring committees, regulators, or researchers. There are sound pragmatic and costs reasons why preliminary attention is paid to social value. Equally, however, funders and other actors require researchers to maximize the value of their research, for example, through open access and/or publication of negative findings. These new milestones represent perfect opportunities to revisit social value considerations; as we have suggested, departure from original promissory appeals to social value can be justified better if we reconceive ‘social’ and ‘value’ in liminal terms. Moreover, we do not advocate a heavy‐handed command‐and‐control to police social value. Rather, we posit a more accurate and socially beneficial way to think about and to demonstrate social value beyond the mere rhetorical or promissory.

### Stakeholder engagement

As a final point, we return to the relationship between this reconfiguration of approaches to social value and the importance of social licence. We do not wish to be read to say that any form of social value generated at any point in the research process necessarily renders this process legitimate. Indeed, we invoke the third usage of social value from our earlier taxonomy in this regard: the essential appeal of social value to protect against exploitative research. There is no better illustration than the classic Tuskegee Syphilis Study example where research ‘subjects’ were denied treatment long after penicillin became available for syphilis, precisely because the overarching objective was the successful on‐going pursuit of the social value in better understanding the progress of a chronic disease.^42^ As we have said, an important consideration in social value is the question: ‘valuable to whom?’ A history of abuses in health research reminds us to qualify our requirement of social value non‐negotiably with the actors involved and affected.

Yet liminality also reminds us about the potentially transformative experience of being involved in health research, *whether for good or ill*. While it is not the case that research participants must necessarily derive benefit or value from the research in which they are involved (indeed, it is usually not the case), their involvement does place them at a central pillar of the research process, and as such we would argue that participants are crucial actors in the construction of the ‘social’ of social value.^43^ Moreover, participant involvement is likely to inform and transform the construction of value from research itself, as a liminal heuristic reminds us. Participants re‐enter an established social order, perhaps transformed by their research experience, and potentially as beneficiaries of the social value of the research itself, but not necessarily in ways hitherto imagined. Further, this way of understanding social value and health research offers new insights into the relation between participant engagement and stakeholder engagement. In a last example, we consider stakeholder engagement and patient engagement in research as an illustration of the five elements of social value proposed above, in particular, spatiality and reliability.

There is a growing realization of the benefits reaped from treating participants as partners in health research. These include more robust research infrastructures, increased trust (and healthy scepticism) in biomedicine, and the development of therapeutics tailored to life with a disease.^44^ A liminal approach to health research requires that we pay attention to *how* such benefits transform throughout the lifecycle of a research project as findings emerge and goals change. In research fields such as rare diseases, where patient involvement in research and development is common, care is needed to maintain the foundational relationship between medical staff, researchers, and patients/participants. In such settings, patients are sometimes both funders and participants of health research; they provide the economic (financial) and epistemic (knowledge and physical bodies) resources needed to make laboratory and clinical research happen. A liminal approach thus requires that participants be engaged throughout the entire research enterprise in a way that maintains the stability of core relationships in a domain characterized by uncertainty and change.

Patient‐supported research provides a unique example for understanding the benefits of a liminal approach to assessments of social value in health research. Opposed to the likes of clinical trials for common disorders, research into rare diseases takes place in the context of tightly knit communities. This produces a unique situation of long‐term co‐dependence where participants and researchers rely on each other to make viable health research for historically neglected conditions. While attempts are underway to rethink how best to assess research outcomes in such settings,^45^ the current emphasis in research ethics on a *preliminary* approach to social value often fails to recognize how research trajectories and stakeholder engagement take place in tandem. In the face of delays and setbacks in research trajectories, or complete refractions in R&D aims, patient collectives (and individuals *qua* trial participants) have been required to reassess their funding priorities. For example, following limited success with the development of gene therapy, and a movement in aim from cure to treatment, the Cystic Fibrosis trust temporarily withdrew funding for the UK Cystic Fibrosis Gene Therapy Consortium, requiring a negotiation between stakeholders in order to secure the future (and thus social value) of the research.^46^ While only one case, the example reveals the benefits of approaching social value as temporally situated, embedded, and open to change.

## Conclusion

The lens of liminality draws attention to the inherent uncertainties of research as well as the various structures (or lack thereof) in which scientific, ethical, and legal norms operate. It focuses our attention on the *processual* nature of health research, with its complex interplay of various actors and factors. If we recast the notion of social value in this light, we can see that it is not that the concept lacks strength or scope, but rather that it can be further exploited as a robust ethical tool. Ultimately, re‐conceptualizing social value through the lens of liminality shows us its potential as a broad and ambitious reference point in at least five key respects, and encourages us to be ever‐mindful that both the ‘social’ and the ‘value’ must be revisited and re‐created iteratively throughout the research lifecycle and by all relevant stakeholders. Once we realize this, we might look for alternative approaches: for example, the creation of more flexible and reflexive governance practices, with feedback loops and iterative forms of collaborative regulation, thus allowing us to unleash the potential of ‘social value’ as a concept.

## Footnotes

1
The Nuremberg Code, n.d. Available at: http://www.hhs.gov/ohrp/archive/nurcode.html [Accessed 11 Apr 2016].

2
M. Habets, J. J.M. van Delden & A.L. Bredenoord. The Social Value of Clinical Research. *BMC Med Ethics* 2014; 15: 66; A. Rid & D. Wendler. A Framework for Risk‐Benefit Evaluations in Biomedical Research. *Kennedy Inst Ethics J* 2011; 21: 141–179. 10.1353/ken.2011.000721696094

3
Department of Health (UK). Governance arrangements for research ethics committees: A harmonised edition 2011 (updated April 2012) para. 3.2.2. Available at: https://www.gov.uk/government/uploads/system/uploads/attachment_data/file/213753/dh_133993.pdfhttps://www.gov.uk/government/uploads/system/uploads/attachment_data/file/213753/dh_133993.pdf [Accessed 11 Apr 2016].

4
Ibid: at para. 3.2.3.

5
CIOMS. Final Draft CIOMS Guidelines. 2015. Available at: http://www.cioms.ch/final_draft_CIOMS_guidelines-10_september_2015-WITH_WATERMARKS.pdf [Accessed 10 Sep 2016].

6
Habets, van Delden, and Bredenoord*, op. cit*. note 2.

7
Ibid.

8

A. Wertheimer . The Social Value Requirement Reconsidered: The Social Value Requirement Reconsidered. Bioethics 2015; 29: 301–308. 2547045010.1111/bioe.12128

9

Council for International Organizations of Medical Sciences and World Health Organization (CIOMS) . 2002 International Ethical Guidelines for Biomedical Research Involving Human Subjects. Geneva: CIOMS; Sec. 8. Available at: http://www.cioms.ch/publications/guidelines/guidelines_nov_2002_blurb.htm [Accessed 10 Sep 2016].

10

E.J. Emanuel , D. Wendler & C. Grady . 2008 An Ethical Framework for Biomedical Ethics In The Oxford Textbook of Clinical Research Ethics, EmanuelE.J. et.al., eds. Oxford and New York: Oxford University Press: 127.

11

D. M. Wenner . The Social Value of Knowledge and International Clinical Research. Dev World Bioeth 2015; 15:76–84. 2619525310.1111/dewb.12037

12
Wertheimer, *op. cit*. note 8, p. 302.

13
Emanuel et al. *op. cit* note 16, p. 127; A. London. A Non‐Paternalistic Model of Research Oversight: Assessing the Benefits of Prospective Review. *J Law Med Ethics* 2012; 40: 942. 10.1111/j.1748-720X.2012.00722.x23289696

14
Wertheimer, *op. cit*. note 8, p. 302.

15
CIOMS, *op. cit*. note 5.

16
A. Van Gennep. 1960. *The Rites of Passage* Chicago: University of Chicago Press; B. Thomassen. 2014. *Liminality and The Modern: Living Through the In‐Between*. Farnham: Ashgate.

17
Van Gennep, *op. cit*. note 16, p. 21.

18
B. Thomassen. 2014. *Liminality and The Modern: Living Through the In‐Between* Farnham: Ashgate; A. Horvath, B. Thomassen& H. Wydra, eds. 2015. *Breaking Boundaries: Varieties of Liminality*. Oxford/New York: Berghahn Books.

19
K. Knorr Cetina. 2009. *Epistemic Cultures: How the Sciences Make Knowledge* Cambridge, MA: Harvard University Press. See also H‐J Rheinberger. 1997. *Toward a History of Epistemic Things. Synthesizing Proteins in the Test Tube*. Stanford, CA: Stanford University Press.

20
Emanuel et al., *op. cit*. note 10.

21
Emanuel et al., *op. cit*. note 10.

22
M‐P. Kieny. 2016. *WHO Research and Development on Zika: Commentary* Available at: http://www.who.int/mediacentre/commentaries/zika-research-development/en/ [Accessed 10 Sep 2016].

23
Rid & Wendler, *op. cit*. note 2.

24
Ibid.

25
Ibid.

26

P. Calain . The Ebola Clinical Trials: a precedent for research ethics in disasters. J Med Ethics 2016: 0:1–6 10.1136/medethics-2016-103474PMC574930727573153

27
WHO, *op. cit*. note 22. p. 2

28
Ibid

29
Calain, *op. cit*. note 27.

30
WHO, *op cit*. note 22. P. 3

31
L.Eckenwiler, J.Pringle, R.Boulanger, and M. Hunt, ‘Real‐time Responsiveness for Ethics Oversight During Disaster Research’, 29:9 *Bioethics* (2015), p. 654. 10.1111/bioe.1219326481207

32

A.H. Kelly & P.W. Geissler . The Value of Transnational Medical Research.” J Cult Econ 2011; 4: 3–10. 2187413510.1080/17530350.2011.535329PMC3158132

33
Ibid.

34
CIOMS, *op. cit*. note 3, Guideline 1.

35
M. Habets et al. *op. cit*. note 2, p. 6.

36
A. Rid & D. Wendler. *op. cit*. note 2, p. 144.

37
A. London, *op. cit*. note 13, p. 942.

38
G. Laurie. Reflexive governance in biobanking: on the value of policy led approaches and the need to recognise the limits of law. Hum Genet (2011) 130: 347. doi:10.1007/s00439-011-1066-x. 10.1007/s00439-011-1066-x21766192

39
G. Laurie, K. Hunter, S. Cunningham‐Burley. Guthrie Cards in Scotland: Ethical, Legal and Social Issues. Report, Scottish Government Social Research. 2013; 4.

40

M. Huckaby Lewis . Lessons from the Residual Newborn Screening Dried Blood Sample Litigation. J Law Med Ethics 2015; 43: 32–35. 2584616010.1111/jlme.12211

41

B. Latour . 2005 Reassembling the Social: An Introduction to Actor‐Network‐Theory. Oxford and New York: Oxford University Press: 1 (emphasis in original).

42

J.H. Jones . 2008 The Tuskegee Syphilis Experiment In The Oxford Textbook of Clinical Research Ethics, EmanuelE.J et al., eds. Oxford and New York: Oxford University Press: 90–93.

43

E.J. Emanuel , D. Wendler , J. Killen , C. Grady . What Makes Clinical Research in Developing Countries Ethical? The Benchmarks of Ethical Research. J Infect Dis 2004; 189(5): 930–937. 1497661110.1086/381709

44

K. Saha & J.B. Hurlbut . Research Ethics: Treat Donors as Partners in Biobank Research. Nature 2011; 478: 312–313. 2201237210.1038/478312a

45

L. Rajmil , L. Perestelo‐Pérez & M. Herdman . Quality of Life and Rare Diseases. Adv Exp Med Biol, 2010; 686: 251–272. 2082445010.1007/978-90-481-9485-8_15

46
This observation is based on one of the author's (in preparation) sociotechnical history of the UK Cystic Fibrosis Gene Therapy Consortium.

